# TGF-β1 promotes cell barrier function upon maturation of corneal endothelial cells

**DOI:** 10.1038/s41598-018-22821-9

**Published:** 2018-03-13

**Authors:** Véronique Beaulieu Leclerc, Olivier Roy, Kim Santerre, Stéphanie Proulx

**Affiliations:** 1 Centre de recherche du Centre hospitalier universitaire (CHU) de Québec – Université Laval, axe médecine régénératrice, Hôpital du Saint-Sacrement, Québec, QC Canada; 2 Centre d’organogénèse expérimentale de l’Université Laval/LOEX, Québec, QC Canada; 3 Département d’Ophtalmologie, Faculté de médecine, Université Laval, Québec, QC Canada

## Abstract

Human corneal endothelial cells (HCECs) easily become fibroblastic-like when cultured, rendering them unsuitable for tissue engineering of the cornea. Transforming growth factor β (TGF-β) could be a key factor in this phenomenon; however, TGF-β is also known to maintain the endothelium in a quiescent state *in vivo*. This work aimed to compare the effects of TGF-β1 on the phenotype of HCECs during the proliferation and maturation phases. Our results show that addition of TGF-β1 during the active proliferation phase produced fibroblastic HCECs and loss of the cell junction markers ZO-1 and n-cadherin, independent from the presence of epidermal growth factor (EGF). By contrast, addition of TGF-β1 in maturation media containing few mitogens led to an endothelial phenotype and functional cell junctions as HCECs developed a high trans-endothelial resistance. Furthermore, addition of AG-1478, an epithelial growth factor receptor inhibitor, enhanced the gain of the endothelial phenotype and cell barrier function. Overall, these results show that TGF-β1 can be used to promote the formation of a typical leaky endothelial barrier during the maturation phase of cultured HCECs. A two-phase culture of HCECs using distinct proliferation and maturation media could also be key for developing ideal HCEC culture conditions.

## Introduction

The corneal endothelium is a monolayer of hexagonal cells that forms a leaky barrier between the corneal stroma and the aqueous humour of the anterior chamber of the eye. The main role of the corneal endothelium is to maintain the corneal stroma in a slightly dehydrated state (termed corneal deturgescence) that is required for optical transparency. The leaky barrier is attributed, at least in part, by the presence of the discontinued tight junction protein ZO-1 around the cells^1^. To counterbalance the influx of liquid into the stroma, endothelial cells actively transport fluid back to the anterior chamber, which is achieved through Na+K+-ATPase pumps and other ion transporters^2^. Diseases that affect this “pump-leak” balance lead to stromal edema and loss of corneal transparency, which results in irreversible vision loss for the patient^3^. To date, corneal transplantation is the only effective treatment to restore vision for patients suffering from endotheliopathies. The high demand of donor corneas led researchers to investigate the feasibility of using *in vitro* expanded corneal endothelial cells (CECs) as alternatives to classic corneal transplantation by means of tissue-engineering or cell injection into the anterior chamber^4–7^.

CECs are arrested in the G1 phase of the cell cycle *in vivo*^8^. However, cells retain the ability to proliferate *in vitro* when stimulated with serum and growth factors^9,10^. Indeed, epidermal growth factor (EGF)^11^, basic fibroblast growth factor (bFGF)^12,13^ and various sera and media formulations have been reported to enable cell expansion of CECs^14–17^. However, CECs can change morphology after repeated passaging, going from an endothelial phenotype (monolayer of hexagonal cells) to a fibroblastic-like phenotype (elongated cells that grow on top of each other). This loss of phenotype, also associated with a loss of functionality in part because of the loss of regular intercellular cell junctions^18^, is attributed to an endothelial-to-mesenchymal transition (EndMT)^19^. Transforming growth factor-β1 (TGF-β1) is a known inducer of EndMT of CECs. It has been shown that addition of TGF-β1 to primate and human CECs induces loss of the endothelial phenotype in a dose-dependent manner^20^. Studies on cat^21^ and bovine^22^ CECs have shown that TGF-β1 induces the expression of abnormal extracellular matrix proteins, such as type I collagen, and also express the stress fiber marker α-SMA. TGF-β1 also changes cell morphology from an endothelial to fibroblastic-like phenotype, processes that are classic signs of EMT. Of interest, EGF has also been shown to interact with TGF-β1 to induce EndMT in some epithelial cells^23,24^.

In the last years, many papers have reported on improving the *in vitro* expansion of CECs to avoid the loss of phenotype. Medium conditioned by NIH-3T3^20^ cells or by bone marrow mesenchymal stem cells (BM-MSCs)^25^ has been shown to promote proliferation of CECs while maintaining their endothelial phenotype. Recently, coating the cell culture surface with basement membrane proteins^26^ and addition of lysophosphatidic acid to the medium as an inducer of proliferation^27^ have also shown the ability to prevent EndMT.

Recent reports have described a dual media approach to expand human CECs for several passages while preventing EndMT^28,29^. This approach consists of using two separate media as follows: one for a proliferation phase and another for a maintenance phase. The dual media approach has also been used in concomitance with Y-26732, an inhibitor of Rho associated coiled-coiled kinase (ROCK)^29^. In this culture method, Y-26732 enhances cell adhesion and overall cell yield throughout passages^29^. Recently, blockage of the TGF-β pathway by SB431542, an inhibitor of type I transforming growth factor receptor (TGFβRI) kinase function, has also been proposed as a way to block EndMT of CECs^20^.

However, TGF-β plays important roles in CEC homeostasis. Indeed, all three TGF-β isoforms (TGF-β1, -β2 and -β3) are physiologically present in the aqueous humour of the anterior chamber^30,31^ and have a regulatory role on CECs^32^. TGF-β1 and - β2 have been shown to block proliferation by suppressing entry into S phase^8,33,34^ via upregulation of the G1-phase inhibitor, p27(Kip1)^35,36^. TGF-β has also been shown to induce migration, rather than proliferation, during wound healing of the corneal endothelium^37,38^. TGF-β also drives development and differentiation of corneal cells derived from the neural crest^39^.

We hypothesized that TGF-β influences the CEC phenotype *in vitro* depending on whether cells are in a proliferating phase or in a confluent “maturing” phase. We also hypothesized that there is a synergistic effect between EGF (mitogen component of the basal medium) and TGF-β1 in inducing EndMT of proliferative CECs. The goal of this study was to optimize the culture conditions for CECs. Our results showed that adding TGF-β1 while CECs are in their maturing phase is beneficial for cell morphology and correct cytolocalization of tight and adherens junction proteins. We thus propose to enhance endothelial morphology by establishing a new two-phase culture media that adds TGF-β1 when CECs have reached confluency. Because maintenance of an endothelial phenotype is essential for functionality, every improvement that can be made to culture conditions will help future discoveries in regenerative medicine.

## Results

### TGF-β1 induces EndMT of proliferating human CECs

**Figure 1:**
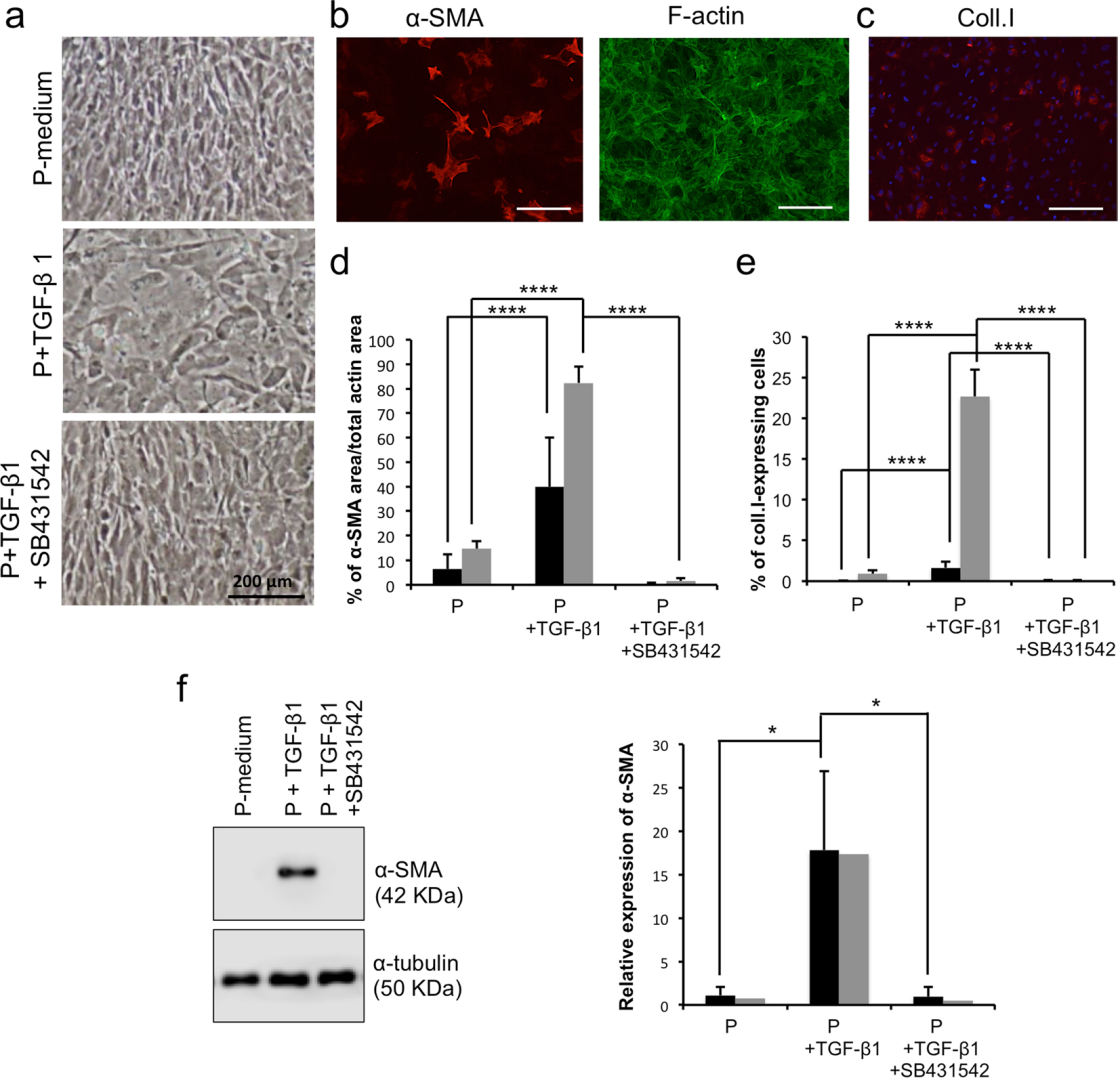
Effects of exogenous TGF-β1 on proliferating human corneal endothelial cells. (**a**) Representative phase contrast images of human corneal endothelial cells (HCEC) grown to confluency in the basal proliferation medium (P) alone (top picture), with TGF-β1 (middle) or with TGF-β1 and SB431542 (bottom). (**b**) Left: Representative micrograph of immunofluorescence detection of α-SMA (red) on HCEC cultured in the basal P-medium+TGF-β1. Right: Immunofluorescent detection of f-actin (green) of the same region. Scale bar: 200 μm. (**c**) Representative micrograph of immunofluorescence detection of type I collagen (red) in HCEC cultured in the P-medium+TGF-β1. Nuclei were countersained using Hoescht 33258 (blue). Scale bar: 200 μm. (**d**–**e**) Image analysis of the immunofluorescent micrographs. The relative expression of α-SMA (d) to f-actin and type I collagen (e) to the number of nuclei was calculated in immunofluorescence micrographs (N = 4, 9 per condition). Results are presented as mean ± S.D. Cell populations in d and e were separated in two subgroups according to their basal protein expression in the proliferation control condition. Low basal expression of α-SMA (d) and coll.I (e) (black bars); high basal expression of α-SMA (d) and coll.I (e) (grey bars). Two-way ANOVA followed by Tukey’s multiple comparison tests were performed. Differences between all conditions in both groups are all statistically significant (p < 0.05). (**f**) Left: Representative Western blot of α-SMA from CECs at confluency. α-tubulin serves as a loading control. Cropped images were displayed and full-length blots are shown in the Supplementary Figure S3. Right: Relative quantification of α-SMA Western blots to the α-tubulin loading control, as described in the materials and methods. Mean of all 4 experiments (1 sample/condition/cell population) were calculated with standard deviation. Cell populations were separated in the same subgroups than in d–e. Two-way ANOVA followed by Tukey’s multiple comparison tests were performed. *p < 0.05; **p < 0.005; ***p < 0.0005; ****p < 0.0001. α-SMA: α-smooth muscle actin; α-tub: α-tubulin; Coll.I: type I collagen.

TGF-β1 has been previously reported to induce a morphological cell change from polygonal to fibroblastic in human CECs^20,38^, which is a characteristic associated with EndMT. In the present proliferating culture conditions, TGF-β1 also induced a change in cell morphology. Figure 1a shows that CECs cultured in the presence of TGF-β1 became less organized and more fibroblastic in appearance compared to the basal proliferation medium (P-medium). Addition of SB431542, an inhibitor of TGFβRI, blocked this effect, and cells retained a similar morphology to those cultured in the absence of TGF-β1 (P- medium).

We next studied the effect of TGF-β1 on the expression of proteins associated with EndMT, such as α-SMA and type I collagen, using immunofluorescence (Fig. 1b–e) at day 0 post-confluency. Interestingly, two types of responses were noted among the different human CEC populations tested. Some CEC populations had low basal levels of α-SMA (<10% of the actin area) and type I collagen (<0.5% of cells) (black bars in Fig. 1d–e), while other populations had a high basal expression of these proteins (α-SMA: >10% of the actin area; type I collagen: >0.5% of cells) (grey bars in Fig. 1d–e). We then decided to separate the CEC populations when analyzing all of the results to verify if they had the same response to TGF-β1 and the various culture conditions. The percentage of coverage by α-SMA (relative to f-actin coverage) was 6.22 ± 5.81% and 14.60 ± 3.06% in the P-medium condition for the low and high basal α-SMA protein expression conditions, respectively, while cells cultured in the presence of TGF-β1 had 39.91 ± 20.15% and 82.36 ± 6.62% of α-SMA coverage for the low and high basal expression populations, respectively (p < 0.0001 for both subgroups). For the low and high basal expression populations, the percentage of type I collagen-expressing cells (Fig. 1e) were 0.02 ± 0.04% and 0.91 ± 0.41% for the P-medium condition, and 1.65 ± 0.77% and 22.66 ± 3.29%, respectively, for the TGF-β condition (p < 0.0001 for low and high basal expression populations). These effects of TGF-β1 were completed counteracted by SB431542 (Fig. 1d–e) in both types of CEC populations (low and a high basal expression of α-SMA and of type I collagen). Western blot analysis of α-SMA (Fig. 1f) confirmed the immunostaining analysis, but the differences between both subgroups were not quantifiable by western blotting for this experiment.

Overall, these results showed that TGF-β1 induces EndMT in actively proliferating human CECs and that the addition of a TGFβRI inhibitor blocks this effect.

### TGF-β1 improves the endothelial phenotype of confluent CECs

CECs are in contact with all three TGF-β isoforms *in vivo* without any consequences on cell phenotype. We next tested if the effect of TGF-β is related to proliferative state (cycling versus non-cycling cells). For this set of experiments, CECs were cultured using a dual media approach by utilizing a proliferation medium (P-medium) during the proliferation phase and a maturation medium (M-medium) once cells reached confluency (Table 1). P-medium consists of Opti-MEM I containing 5 ng/ml epithelial growth factor (EGF), chondroitin sulfate, ascorbic acid and 8% fetal bovine serum. The M-medium consists only of Opti-MEM I and 8% fetal bovine serum. A similar dual media approach has been reported to be optimal for CEC cell expansion^28^.

**Table 1:** Cell culture conditions during proliferation and maturation of HCEC.

Proliferation medium (P)	Maturation medium (M)
P	n/a
P + TGF-*β*1 2 ng/ml	n/a
P + TGF-*β*1 2 ng/ml + SB431542 10 µM	n/a
P + TGF-*β*1 2 ng/ml + AG-1478 1 µM	n/a
P	P
P	M
P	M + TGF-*β*1 2 ng/ml
P	M + TGF-*β*1 2 ng/ml + SB431542 10 μM
P	M + TGF*β*1 2 ng/ml + EGF 5 ng/ml
P	M + TGF*β*1 2 ng/ml + EGF 5 ng/ml + AG-1478 1 µM
P	M + TGF*β*1 2 ng/ml (1–7 DPC) + M (8–14 DPC)

DPC: days post-confluency; EGF: epidermal growth factor; HCECs: human corneal endothelial cells; TGF-β1: transforming growth factor β-1.

**Figure 2:**
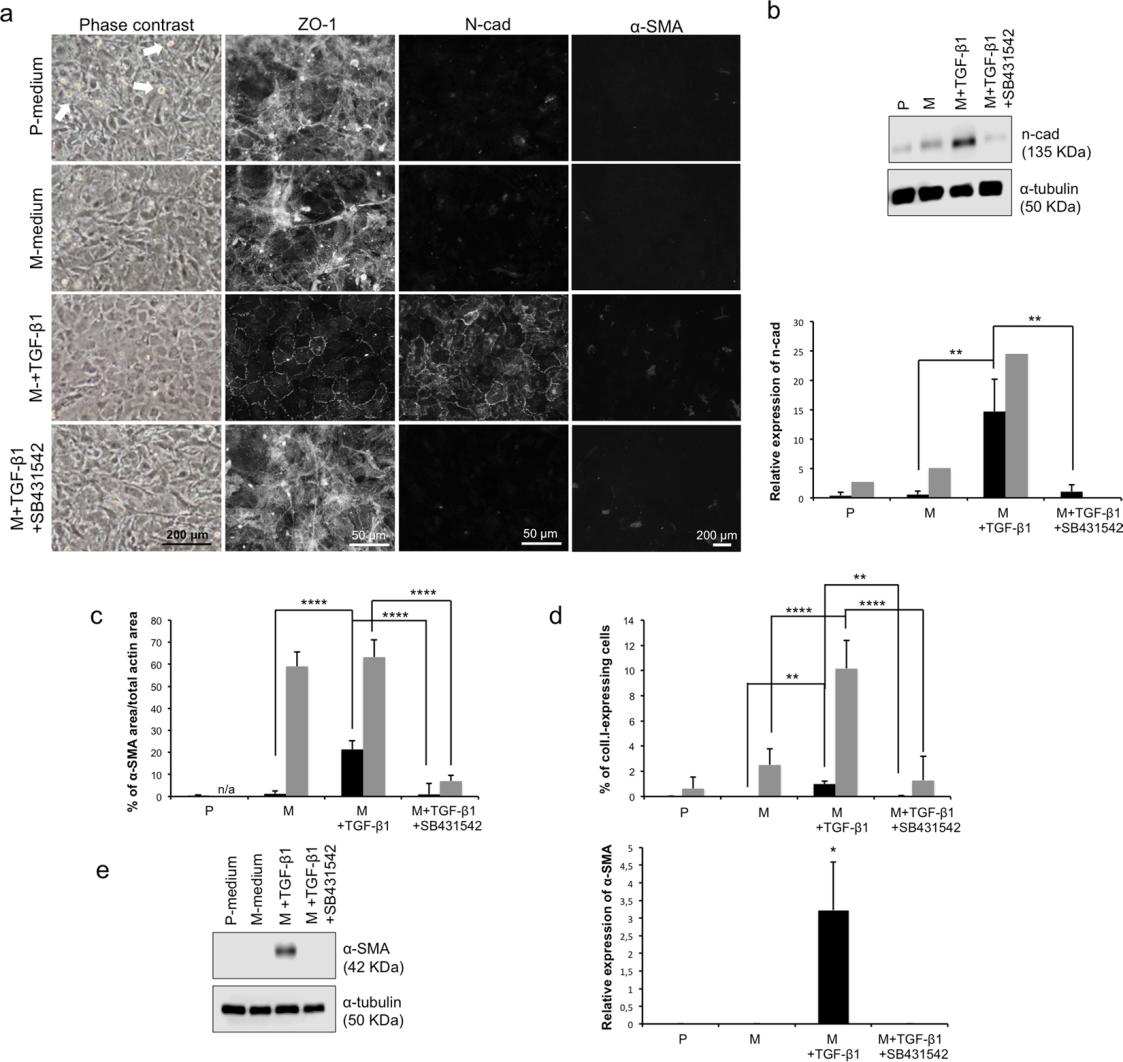
Effects of TGF-β1 on the phenotype of confluent human corneal endothelial cells (HCEC). (**a**) Phase contrast images (left column) and immunofluorescence detection of ZO-1 (middle column), N-Cadherin (middle column) and α-SMA (right column) of CECs cultured in the basal proliferating medium (P-medium) until confluency, then kept for 7 days in the same P-medium, or switched in the basal maturation medium alone (M-medium) or in M+TGF-β1 or in M+TGF-β1+SB431542. White arrows point floating dead cells. A population with low basal α-SMA is represented. (**b**) Representative Western blot of n-cadherin from HCEC at 7 days post-confluency (top). α-tubulin served as a loading control. Cropped images were displayed and full-length blots are shown in the Supplementary Figure S4a. Bottom: Relative quantification of the n-cadherin western blots to the α-tubulin loading control, as described in the materials and methods. Mean of all 4 experiments (1 sample/condition/cell population) were calculated with standard deviation. Cell populations were separated in two subgroups according to their basal protein expression of endothelial-to-mesenchymal transition markers in the proliferation control condition. Low basal expression of α-SMA and coll.I (black bars); high basal expression of α-SMA and coll.I (grey bars). Two-way ANOVA followed by Tukey’s multiple comparison tests were performed. (**c**–**d**) Analysis of immunofluorescence micrographs for endothelial-to-mesenchymal transition markers at 7 days post-confluency. The relative expression of α-SMA (c) to f-actin and type I collagen (d) to the number of nuclei was calculated in immunofluorescence micrographs (9 per condition). Results are presented as mean ± S.D. Cell populations in c and d were separated in two subgroups according to their basal protein expression in the proliferation control condition. Low basal expression of α-SMA (c) and coll.I (d) (black bars); high basal expression of α-SMA (c) and coll.I (d) (grey bars). Two-way ANOVA followed by Tukey’s multiple comparison tests were performed. (**e**) Left: Representative Western blots of α-SMA from CECs at 7 days post-confluency. α-tubulin serves as a loading control. Cropped images were displayed and full-length blots are shown in the Supplementary Figure S4b. Right: Relative quantification of the α-SMA to the α-tubulin loading control, as described in the materials and methods. Mean of 2 experiments (1 sample/condition/cell population) were calculated with standard deviation. Cell populations were separated in the same subgroups than in b-d. One-way ANOVA followed by Tukey’s multiple tests were performed. M+TGF-β1 condition was significantly higher than all other conditions. *p < 0.05; **p < 0.005;***p < 0.0005; ****p < 0.0001. α-SMA: α-smooth muscle actin; α-tub: α-tubulin; Coll.I: type I collagen; n-cad: n-cadherin.

To verify if the addition of TGF-β1 to the medium has a different impact on CECs when added to confluent cells rather than during proliferation, cells were grown to confluency in P-medium and then maintained at post-confluency for 7 days in M-medium alone, M-medium with TGF-β1, or M-medium with TGF-β1 and SB431542. Cell morphology was first observed using phase-contrast microscopy. Figure 2a shows that CECs matured without TGF-β1 (second row) presented a higher amount of elongated cells than those that matured in the presence of TGF-β1. Indeed, addition of TGF-β1 to the maturation medium (third row) enhanced the endothelial morphology of CECs as they became a uniform monolayer of small polygonal cells after 7 days of maturation. Adding SB431542 (last row) blocked the effect of TGF-β1 as indicated by similar cell morphology of CECs cultured in the presence of both TGF-β1 and SB431542 to those cultured without TGF-β1. Finally, cells that were maintained in P-medium after confluency adopted a fibroblastic morphology and had a high number of floating dead cells (white arrows in Fig. 2a, first row), demonstrating that culturing cells in proliferation media once they reached confluency should be avoided for cell expansion of CECs. It is worth noting that the presence of TGF-β1 in the maturation medium significantly improved the circularity of the cells when compared to the P- and M-Medium conditions, except in the condition where SB431542 was also present. These results are available online in Supplementary Figure S2.

The presence of TGF-β1 in the maturation phase also induced a better cytolocalization of ZO-1 at the cell junctions and a higher expression of n-cadherin compared to the P- and M-medium conditions (Fig. 2a). The gain in correct cytolocalization of intercellular junction proteins following the addition of TGF-β1 was counteracted by the addition of SB431542 (last row) as the expression and localization of ZO-1 and n-cadherin decreased to levels similar to the P- and M- medium conditions. Western blot analysis for n-cadherin (Fig. 2b) confirmed the immunostaining results.

Analysis of the ratio of the area covered by α-SMA to the area covered by total actin in immunofluorescence micrographs at day 7 post-confluency (Fig. 2a–c) showed that CECs in the lower expression group (black bars) expressed a higher amount of α-SMA when exposed to exogenous TGF-β1 during the maturation phase (1.95 ± 1.25% vs. 21.25 ± 3.93%; p < 0.0001). Analysis of the percentage of type I collagen-expressing cells at day 7 post-confluency (Fig. 2d) showed similar tendencies in both low (black bars) and high basal expression of the protein (grey bars) (0.00 ± 0.00% and 2.51 ± 1.27% vs. 0.97 ± 0.27% and 10.17 ± 2.25%; p < 0.005 for both low and high basal expression). The expression of these EndMT markers was completely blocked by SB431542. Western blot analysis of α-SMA (Fig. 2e) confirmed these results as α-SMA expression completely disappeared when SB431542 was present in the culture medium. This effect is similar to the effects of TGF-β1 on the expression of fibroblastic proteins during proliferation (see Fig. 1). These findings showed that TGF-β1 has different effects on the phenotype of confluent cultured HCECs as follows: a negative effect (expression of fibroblastic proteins α-SMA and type I collagen) and a positive effect (enhancement of the endothelial morphology and expression of cell-junction proteins ZO-1 and n-cadherin).

### Use of TGF-β as a punctual inducer of cell junctions

To test if TGF-β1 is a punctual inducer of cell junction proteins without inducing expression of EndMT markers, confluent CECs were cultured in the presence of exogenous TGF-β1 in the basal maturation medium (M-medium) for 7 days followed by additional culture for 7 days in M-medium without TGF-β1.

**Figure 3:**
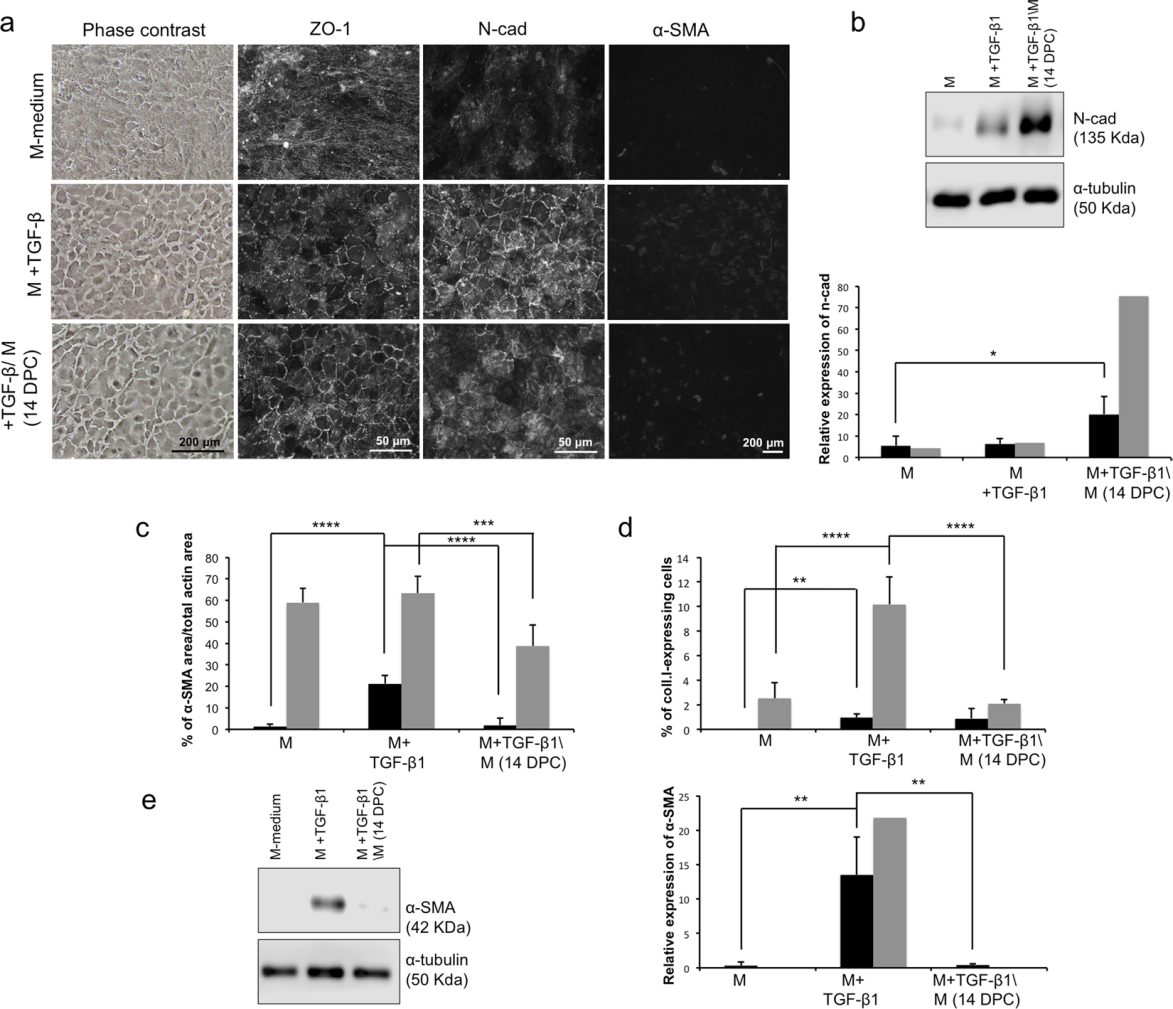
Effect of TGF-β1 on the expression of cell junction proteins in confluent cultures human corneal endothelial cells (HCEC). (**a**) Phase contrast images (left column) and immunofluorescence detection of ZO-1 (middle column), N-Cadherin (middle column) and α-SMA (right column) of CECs cultured in the basal proliferating medium until confluency, then switched for 7 days in the basal maturation medium alone (M) or with M+TGF-β1, or M+TGF- β1 for 7 days followed by a period of 7 days without TGF-β1 (14 days post-confluency). A population with low basal α-SMA expression is represented. (**b**) Top: Representative Western blot of n-cadherin from HCEC at 7 days post-confluency in the basal maturation medium (M), with TGF-β1 (+TGF-β1) or 14 days post-confluency (+TGF-β1/M). α-tubulin served as a loading control. Cropped images were displayed and full-length blots are shown in the Supplementary Figure S5a. Bottom: Relative quantification of the n-cadherin Western blots to the α-tubulin loading control, as described in the Materials and Methods. Mean of all 4 experiments (1 sample/condition/cell population) were calculated with standard deviation. Two-way ANOVA followed by Tukey’s multiple comparison tests were performed. Cell populations were separated in the same subgroups described in c–d. **(c**–**d**) Analysis of immunofluorescence micrographs for endothelial-to-mesenchymal transition markers. The relative expression of α-SMA (c) to f-actin and type I collagen (d) to the number of nuclei was calculated in immunofluorescence micrographs (9 per condition). Results are presented as mean ± S.D. Cell populations in c and d were separated in two subgroups according to their basal protein expression in the proliferation control condition. Low basal expression of α-SMA (c) and coll.I (d) (black bars); high basal expression of α-SMA (c) and coll.I (d) (grey bars). Two-way ANOVA followed by Tukey’s multiple comparison tests were performed. (**d**–**e**) Left: Representative Western blots of α-SMA from CECs at 7 days post-confluency in a basal maturation medium (M), with TGF-β1 (+TGF-β1) or 14 days post-confluency (+TGF-β1/M). α-tubulin serves as a loading control. Cropped images were displayed and full-length blots are shown in the Supplementary Figure S5b. Right: Relative quantification of the α-SMA Western blots to the α-tubulin loading control as described in the Materials and Methods. Mean of all 4 experiments (1 sample/condition/cell population) were calculated with standard deviation. Cell populations were separated in the same subgroups than in c-d. Two-way ANOVA followed by Tukey’s multiple comparison tests were performed. *p < 0.05; **p < 0.005; ***p < 0.0005; ****p < 0.0001 α-SMA: α-smooth muscle actin; α-tub: α-tubulin; Coll.I: type I collagen; DPC: days post-confluency; n-cad: n-cadherin; TGF-β1: Transforming growth factor β1.

Addition of exogenous TGF-β1 to the maturation medium improved the morphology of HCECs compared to M-medium alone (Fig. 3a, first column), thereby reproducing the results in Fig. 2. Cells retained an endothelial morphology after the 7 day period of culture without TGF-β1. However, this TGF-β1 removal partially decreased the expression of n-cadherin at cell junctions as they were less uniform (Fig. 3a, third column, 14 DPC). ZO-1 (Fig. 3a, second column) was correctly expressed at cell junctions in a polygonal pattern in both TGF-β1 conditions compared to M-medium alone.

Western blot analysis for n-cadherin (Fig. 3b) showed a higher expression at 14 days post-confluency than at 7 days post-confluency (with or without TGF-β1). This effect could be explained by the longer culture period, which favors formation of cell junctions because of the higher density of the cells^40^.

Following the 7 day TGF-β1 treatment, cells expressed α-SMA and type I collagen (Fig. 3c,d and e) as expected (Fig. 2b,c and d). However, the expression levels became similar to that of the basal maturation medium (M-medium) after the second set of 7 days of maturation without TGF-β1. Overall, these results showed that the gain of EndMT markers following addition of TGF-β1 to confluent CECs is transient and reversible and that expression of cell junction proteins is mostly preserved even after 7 days without TGF-β1.

### Evaluation of the combined effects of TGF-β and EGF on the phenotype of cultured HCECs

**Figure 4:**
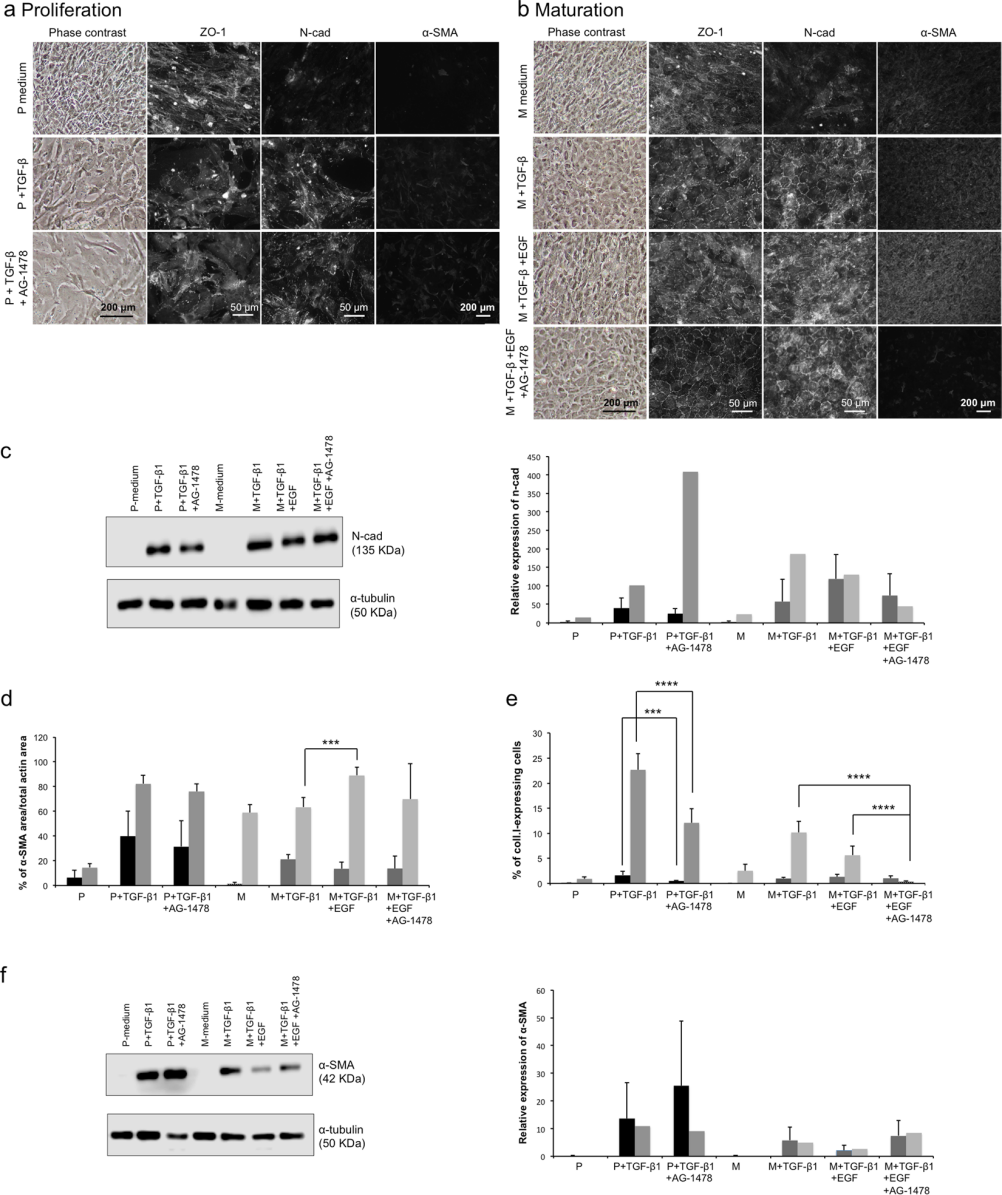
Evaluation of the combined effects of TGF-β1 and EGF on the morphology and the phenotype of corneal endothelial cells in the proliferation and the maturation phases. (**a**) Proliferation phase: Phase contrast images (left column), immunofluorescence detection of ZO-1 (second column), N-Cadherin (middle column) and α-SMA (right column) of CECs cultured in a proliferation medium alone (P) or supplemented with TGF-β1 or a combination of TGF-β1 and the EGFR inhibitor AG-1478. Note that the proliferation medium contains 5 ng/ml of EGF. (**b**) Maturation phase: CECs were cultured in the proliferation medium until confluency then cultured for 7 days in the maturation medium alone (M) or supplemented with TGF-β1 or a combination of TGF-β1 and EGF, or a combination of TGF-β1, EGF and AG-1478. Note that the basal maturation medium does not contain EGF. (**c**) Left: Representative Western blot of n-cadherin from CECs at confluency (P-conditions) or at 7-days post-confluency (M-conditions). α-tubulin served as a loading control. Cropped images were displayed and full-length blots are shown in the Supplementary Figure S6a. Right: Relative quantification of the n-cadherin Western blots to the α-tubulin loading control, as described in the Materials and Methods. Mean of all 4 experiments (1 sample/condition/cell population) were calculated with standard deviation. Cell populations were separated in the same subgroups of high and low basal expression of α-SMA and coll.I (described in d-e). Two-way ANOVA followed by Tukey’s multiple comparison tests were performed. (**d**–**e**) Analysis of immunofluorescence micrographs for endothelial-to-mesenchymal transition markers. The relative expression of α-SMA (d) to f-actin and type I collagen (e) to the number of nuclei was calculated in immunofluorescence micrographs (9 per condition). Results are presented as mean ± S.D. Cell populations in d and e were separated in two subgroups according to their basal protein expression in the proliferation control condition. Low basal expression of α-SMA (d) and coll.I (e) (black bars); high basal expression of α-SMA (d) and coll.I (e) (grey bars). Two-way ANOVA followed by Tukey’s multiple comparison tests were performed. (**f**) Left: Representative Western blot of α-SMA (e) from CECs at confluency (P conditions) or at 7-days post-confluency (M-conditions). α-tubulin served as a loading control. Cropped images were displayed and full-length blots are shown in the Supplementary Figure S6b. Right: Relative quantification of the α-SMA Western blots to the α-tubulin loading control, as described in the Materials and Methods. Mean of all 4 experiments (1 sample/condition/cell population) were calculated with standard deviation. Cell populations were separated in the same subgroups than in d-e. Two-way ANOVA followed by Tukey’s multiple comparison tests were performed. *p < 0,05; **p < 0.005; ***p < 0.0005; ****p < 0.0001. α-SMA: α-smooth muscle actin; α-tub: α-tubuline; Coll.I: type I collagen; EGF: Epidermal growth factor; n-cad: n-cadherin; TGF-β1: Transforming growth factor β1.

Since EGF is a mitogen that is commonly used in culture media for CECs (including ours), we tested the possible synergistic effect between EGF and TGF-β1 in inducing EndMT, in both the proliferation (Fig. 4a) and the maturation (Fig. 4b) phases.

#### Proliferation phase

Addition of TGF-β1 to the P-medium (which contains EGF; Table 1) changed the morphology of the cells. The cells became larger, elongated and less uniform than those cultured in P-medium without TGF-β (P+EGF) (Fig. 4a) as shown in Figs 1 and 3. When EGFR was blocked by adding AG-1478, cell morphology also changed. Cells became larger and grew less on top of each other. The addition of TGF-β1 (with or without AG-1478) also generated the expression of n-cadherin at the cell membrane. The gain of n-cadherin expression following addition of TGF-β1 was confirmed using western blotting (Fig. 4c).

Expression of the EndMT marker, α-SMA (Fig. 4a and d), was similar in the presence of AG-1478 compared to TGF-β. These results were confirmed by western blot analysis (Fig. 4f). Adding AG-1478 decreased the percentage of type I collagen-expressing cells (Fig. 4e; p = 0.0002 and p < 0.0001 for low (black bars) and high (grey bars) basal expression, respectively).

#### Maturation phase

The M-medium does not contain EGF (Table 1). Compared to CECs that matured in M-medium with TGF-β1 (Fig. 4b, second row), addition of EGF and TGF-β1 (Fig. 4b, third row) generated a less uniform cell monolayer. Adding AG-1478 to this mixture (M-medium+TGF-β+EGF+AG1478) helped the endothelial morphology as cells had a better cytolocalization of ZO-1 at the cell border with less ZO-1 expression in the cytoplasm (Fig. 4b). N-cadherin cytolocalization and level of expression was enhanced in the presence of TGF-β1, and similarly expressed regardless of EGF or AG-1478 supplementation (Fig. 4b,c).

Analysis of the α-SMA immunostaining results show that the percentage of α-SMA cell coverage at day 7 post-confluency (Fig. 4d) increased in the presence of TGF-β1 during maturation for the low basal α-SMA group (dark grey bars). The levels remained similar with the addition of EGF and/or AG-1478 in both groups with the exception of a 1.4-fold higher α-SMA expression when EGF was added to the M-medium+TGF-β1 (M+TGF-β1+EGF) compared to the M-medium+TGF-β1 condition for the population with a higher basal expression of α-SMA (pale grey bars, 63.27 ± 7.92% vs. 89.03 ± 6.70%; p = 0.0004). These results were similar in western blot analysis, except for the M+TGF-β1+EGF condition where the α-SMA expression tended to be lower than in the M+TGF-β1 and M+TGF-β1+EGF+AG-1478 conditions (Fig. 4f).

The percentage of type I collagen-expressing cells at day 7 post-confluency (Fig. 4e) was near zero when AG-1478 was added to the maturation medium for the population with a higher basal expression of type I collagen (pale grey bars), representing a diminution when compared to the TGF-β1 alone and TGF-β1+EGF medium for the same population (p < 0.0001). These results suggested that EGF and TGF-β1 have no significant synergistic effect in inducing EndMT of proliferating and maturating HCECs. However, blocking EGFR helps to acquire an endothelial phenotype during maturation.

### Improvement of cell culture conditions: Use of TGF-β1 and AG-1478 in a two-phase culture

**Figure 5:**
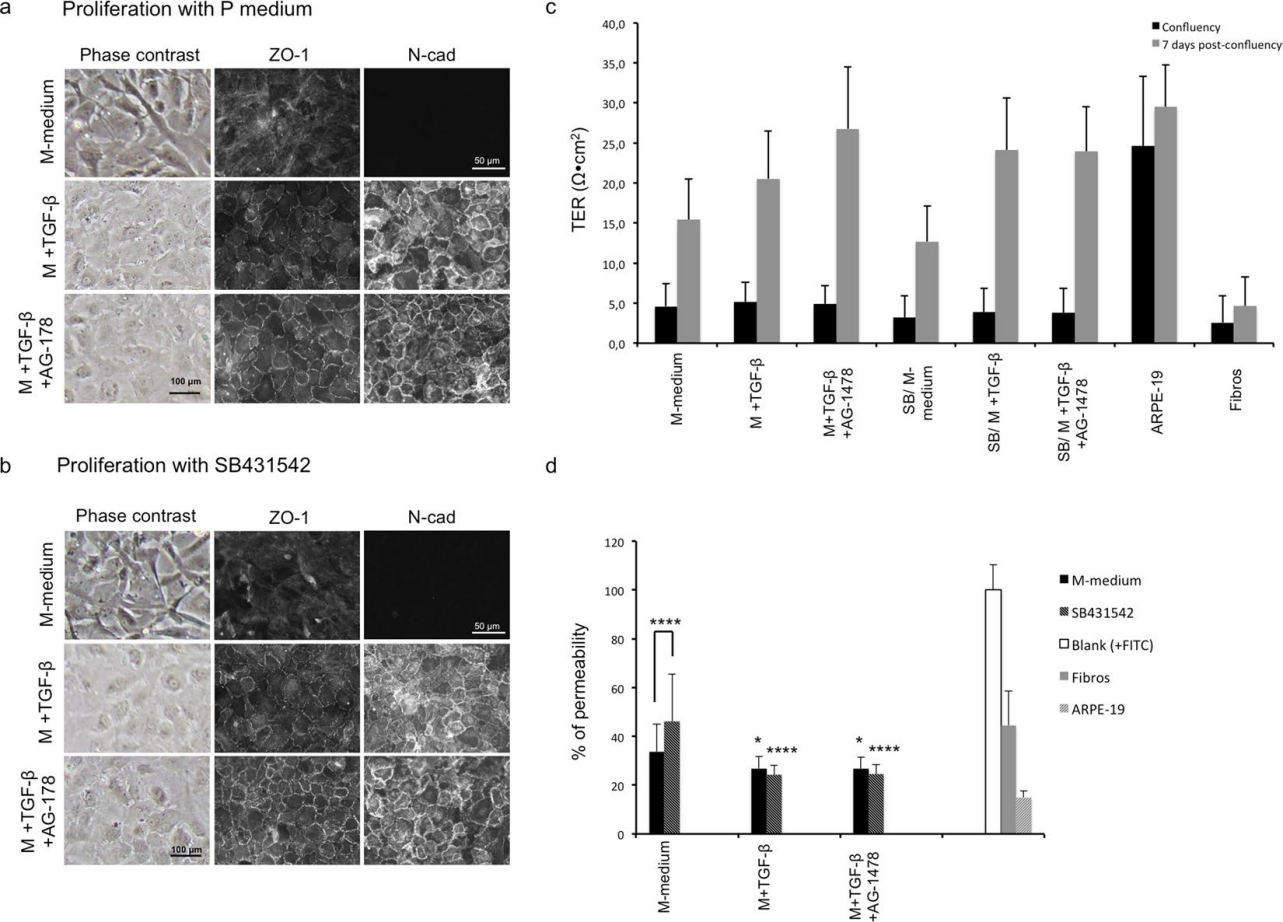
Improvement of cell culture conditions: use of TGF-β1 and AG-1478 in a 2-phases culture. (**a**) Phase contrast images (left column), immunofluorescence detection of ZO-1 (middle column) and N-Cadherin (right column) of CECs grown on cell culture inserts. Morphology (phase contrast images) was assessed on CECs grown in a 12-well culture plate in the same conditions as the culture inserts. Cells were cultured in a basal proliferation medium (P-medium) until confluency. Medium was then switched to a maturation medium (M-medium) alone or supplemented with TGF-β1 or TGF-β1 and AG-1478, for 7 days post-confluency. Results at 7 days post-confluency are presented. (**b**) HCECs were grown in the P-medium containing SB431542 (10 μM) until confluency, then switched in the M-medium alone or supplemented with TGF-β1 or TGF-β1 and AG-1478, for 7 days post-confluency. The same analyses as in (**a**) were conducted. (**c**) Trans-endothelial resistance (TER) at confluency (black bars) and 7 days post-confluency (grey bars) of HCEC cultured on cell culture inserts. They proliferated in the P-medium then were switched to the M-Medium for maturation, or they proliferated in the P-medium+SB431542 before maturation (SB/M-medium). The maturation period was done in the M-medium alone or with TGF-β1 or TGF-β1 and AG-1478. TER was calculated with 15 measurements for each condition. Measurements were normalized with an empty insert containing the same medium. ARPE-19 and corneal fibroblasts served as positive and negative controls for the formation of a barrier. One-way ANOVA followed by Tukey’s multiple comparison tests were performed. N = 3 cell populations (3 culture inserts/condition, 5 measurements/insert), mean ± standard deviation. (**d**) Trans-endothelial permeability to Dextran-FITC (4 KDa) at 7 days post-confluency of HCEC cultured in the same conditions as in (**c**). Mean of the measurements was calculated and normalized with the fluorescence from an empty insert. ARPE-19 and corneal fibroblasts served as positive and negative controls for the formation of a cell barrier. One-way ANOVA followed by Tukey’s multiple comparison tests were performed. N = 3 cell populations (5 samples per condition), mean ± standard deviation. *p < 0,05; **p < 0.005; ***p < 0.0005; ****p < 0.0001; when directly over the bar, it is compared to their respective M-medium or SB/M-medium controls. n-cad: n-cadherin; fibros: corneal fibroblasts; SB: SB431542; TER: trans-endothelial resistance; TGF-β1: Transforming growth factor β1.

To test if culture conditions of CECs could be improved by a strategic use of SB431542, TGF-β1 and AG-1478, we cultured cells in standing inserts (for trans-endothelial resistance and permeability measurements) and in plastic culture wells (for morphology analysis). Half of the cells proliferated in the P-medium (Fig. 5a) and the other half in the P-medium supplemented with SB431542 (Fig. 5b). After confluency, cells in both conditions were cultured for 7 days in the M-medium, M-medium+TGF-β1, or M-medium+TGF-β1+AG-1478.

Influence of the proliferation media on the final CEC phenotype: After the 7 day maturation period, cell morphology and correct cytolocalization of ZO-1 and n-cadherin were similar whether they proliferated with or without SB431542 (Fig. 5a and b). However, when analyzing trans-endothelial resistance (TER) at confluency (Fig. 5c), the TER of HCECs proliferated with SB431542 tended to be lower than the one from cells proliferated without SB431542 in all three conditions. This trend was also observed at day 7 post-confluency. The TER from cells that proliferated with SB431542 was only higher than that of cells proliferated in P-medium in the TGF-β1 condition at day 7 post-confluency (+TGF-β vs. SB/+TGF-β). However, these results were not statistically significant. Permeability to Dextran-FITC 4 KDa (Fig. 5d) 7 days post-confluency was affected differently with SB431542 treatment in one proliferation condition as it was higher than that in cells proliferated without SB431542 (M-medium vs SB/M-medium, grey bars) (p < 0.0001).

Cells that matured in M-medium were more elongated and fibroblastic-like than cells matured with TGF-β1±AG-1478, which were more regular and endothelial-like as indicated by the morphology shown in phase contrast images. Expression and localization of ZO-1 and n-cadherin were the most apparent in the TGF-β1+AG-1478 conditions, while cells cultured in M-medium expressed little ZO-1 and no n-cadherin, which agreed with the results in Fig. 4b-c.

Influence of the maturation media on the final CEC phenotype: An enhanced expression of ZO-1 and n-cadherin at cell junctions was observed when cells were matured in M-medium supplemented with TGF-β1+AG-1478 compared to cells cultured in M-medium with or without TGF-β1 (Fig. 5a and b). TER analysis (Fig. 5c) also showed that maturation is necessary to acquire a higher TER regardless of the factors added to the maturation medium.

Remarkably, the enhanced ZO-1 and n-cadherin expression in the presence of TGF-β1 or TGF-β1+AG1478 correlated with a tendency of higher trans-endothelial resistance (Fig. 5c) than with M-medium at day 7 post-confluency (grey bars). Expression of cell junction proteins in the conditions that matured with TGF-β1 and TGF-β1 plus AG-1478 also correlated with a lower permeability (Fig. 5d) of the cell monolayer to Dextran-FITC 4 KDa compared to cells cultured with M-medium alone.

Addition of TGF-β1 and AG-1478 to the maturation media would therefore be an efficient way to improve the endothelial phenotype of HCECs in two-phase culture of HCECs with or without addition of SB431542 to the proliferation media.

## Discussion

This study reported that TGF-β1 not only induces EndMT of proliferating HCECs but also improves the endothelial phenotype and cell barrier function when added during the maturation phase. Moreover, blocking EGFR, combined with the addition of TGF-β1 to basal maturation media, further improves the endothelial phenotype. To our knowledge, this phenomenon has not been previously shown for this type of cells.

TGF-β1 is an inducer of mesenchymal transition of cultured HCECs^20^. In a 2013 study, Okumura *et al*. showed that cultured primate corneal endothelial cells naturally lose their endothelial phenotype, express abnormal extracellular matrix proteins, such as type I collagen and fibronectin, and also express the stress fiber marker α-SMA. These researchers found that signaling from the TGF-β pathways (phosphorylation of Smad2, p38 and ERK 1/2) is implicated in EndMT of the cultured CECs. Concurrently, they demonstrated that blockage of type I TGF-β receptor kinase activity by SB431542 prevents EndMT of cultured CECs and improves the function-related ZO-1 and Na^+^K^+^-adenosine triphosphatase (Na^+^K^+^-ATPase) protein expression. Our results showed that TGF-β1 indeed induced EndMT of HCECs during the proliferation phase as cells expressed fibroblastic-like proteins (α-SMA and type I collagen) and that blocking the TGF-β1 pathway by SB431542 greatly reduced their expression. However, when used during the maturation phase of HCECs, TGF-β1 and SB431542 demonstrated contradictory roles. TGF-β1 became useful to the endothelial phenotype of confluent HCECs, and SB431542 blocked its positives effects. TGF-β1 promoted the correct cytolocalization of the cell junction proteins, ZO-1 and n-cadherin (localization at the cell junctions instead of the cytoplasm), resulting in the formation of a monolayer of endothelial cells rather than unorganized fibroblastic cells. Recently, a team reported that the continuous use of SB431542 reduces the number of HCECs recovered at P0 and induces morphological changes, rendering them unsuitable for clinical use^41^.

The dual role of TGF-β isoforms is well documented. On the one hand, TGF-β isoforms (especially TGF-β1) are essential for the embryonic development and for physiological functions in the adult, such as angiogenesis and immunity. They are also known as anti-proliferative cytokines and tumor suppressors^42^. On the other hand, TGF-β isoforms can be involved in many aspects of cancer progression, such as proliferation, angiogenesis and metastasis, according to the mean of epithelial-to-mesenchymal transition (EMT)^43^. TGF-β2 is known to help maintain the integrity of the corneal endothelium *in vivo* by inhibiting the G1/S phase-transition^35^ and promoting migration^37^. However, the role of TGF-β on the formation of cell junctions has not been reported before in CECs.

TGF-β signaling is also highly influenced by the cellular or environmental context^44^. Interesting studies on renal tubular epithelial cells have clearly demonstrated the context-dependent roles of TGF-β on inducing EMT^45,46^. For example, it has been shown that disruption of cell-cell contacts (by wounding, contact disassembly by Ca^2+^ removal or subconfluency) is necessary for TGF-β1 to induce EMT. Furthermore, adding TGF-β1 to confluent monolayers does not generate EMT of renal tubular epithelial cells^45^. It has also been reported that TGF-β1 and loss of cell-cell contact have a synergic effect on the induction of the SMA gene promoter and that β-catenin has a role in the regulation of the promoter in these conditions. In addition, these researchers noticed that the SMA promoter is activated under normal conditions without exogenous TGF-β1 in subconfluent cultures, and they further characterized this mechanism by showing that the Rho-ROCK pathway also regulates the SMA promoter in contact-injured TGF-β1-treated tubular cells^46^. These findings are similar to the ones reported by Scheffer C. G. Tseng’s team as they found that disrupting cell-cell junctions of HCECs with EDTA followed by a treatment with bFGF causes EndMT and that this effect is amplified by addition of TGF-β1 to the culture medium^38^. Cells treated with TGF-β1 lose their typical junctional n-cadherin expression to a nuclear expression of n-cadherin. Our results also showed an important cell contact-dependent role of TGF-β1 on inducing EndMT. Subconfluent cells that lack cell-cell junctions were more prone to lose their endothelial morphology, express α-SMA, express type I collagen and have irregularly distributed cell junction proteins (Figs 1 and 4). However, with confluent cells, TGF-β1 greatly improved the morphology and expression of ZO-1 and n-cadherin at the cell junctions. Studies have also shown the importance of having a high cell density when passaging CECs^40^ to rapidly regain the expression of cell-cell junction proteins lost by the use of trypsin-EDTA^47^ or other cell junction-disrupting agents. High cell seeding density also decreased cell expansion in our model as CECs attained confluency faster allowing an earlier start for the 7 day TGF-β treatment.

Contrary to the findings on tubular epithelial cells^45,46^, confluent HCECs still expressed α-SMA in presence of TGF-β1 (Fig. 3), but this expression, along with type I collagen expression, was highly or completely reduced after withdrawal of TGF-β1 for 7 additional days without affecting the endothelial phenotype. α-SMA expression in confluent cells could be explained by the fact that cells were not 100% confluent. A few more days of culture may let the cells establish better cell junctions, and the addition of TGF-β1 may induce less α-SMA expression as indicated in tubular epithelial cells^48,49^. It is worth mentioning that α-SMA did not affect the function of the cells as cells cultured with TGF-β1 had higher trans-endothelial resistance and lower permeability than control cells (Fig. 5c and d).

Some work in other epithelial cell types has shown a synergistic effect between TGF-β and EGF in inducing oncogenic properties^24^, such as invasion and EMT^23^. Thus, we wanted to verify if EGF and TGF-β1 could exert the same effect on HCECs. A synergy between these growth factors could explain, in part, the EndMT induced by TGF-β1 in the proliferation phase, as EGF is only present in the proliferation medium used in this study. However, this hypothesis was not confirmed by our results. We observed small changes in cell morphology (Fig. 4a) and in the expression of EndMT markers (Fig. 4c and d) when EGFR was blocked in the proliferation phase although these results were not sufficient to prove that the interaction between EGF and TGF-β1 is a key factor in the EndMT of proliferating HCECs. However, when blocking EGFR in the maturation phase, we observed that it benefitted the morphology of the cells and the localization of ZO-1 and n-cadherin at the cell junctions when compared to TGF-β1 alone (Fig. 4b). In ARPE-19 cells, TGF-β1 alone induces EMT without Wnt signaling^48^. Interestingly, TGF-β1, in synergy with EGF and FGF-2, has been shown to induce EMT and Wnt signaling when contact inhibition is disrupted in ARPE-19 cells^48^. When Wnt signaling is activated, β-catenin, a component of adherens junctions, is not degraded and is translocated to the nucleus, thereby regulating genes related to proliferation and EMT^49^. β-catenin is liberated in the cytoplasm when cell junctions are mechanically or chemically disrupted (EDTA, trypsin, and Ca^2+^ removal). EGF, TGF-β1 and other mitogens present in the proliferation media could then be involved in the activation of the Wnt pathway and contribute to corneal EndMT. In confluent HCECs, adherens junctions are reassembled, indicating that there is less β-catenin in the cytoplasm that can act as an EndMT inducer. This phenomenon remains to be explored in our culture conditions, but it still suggests that confluence is a central determinant of the TGF-β1 effect on HCECs.

To further characterize the effects of TGF-β1 and AG-1478 on the function of HCECs in a two-phase culture, we used trans-endothelial resistance and permeability to Dextran-FITC 4 kDa in optimized conditions. Blocking the TGF-β pathway using SB431542 during the proliferation phase did not help in the formation of a mature and functional endothelium (Fig. 5). The results showed that adding TGF-β1 and AG-1478 to the maturation medium promotes the formation of a functional monolayer as they promoted a higher trans-endothelial resistance and a lower permeability to Dextran-FITC when compared to cells cultured in control maturation media. TER values measured 7 days post-confluency ranged from 12.68 (SB/M-medium condition) to 26.70 Ω∙cm^2^ (TGF-β1+AG-1478 condition) (Fig. 5c). These values are similar to those obtained by other researchers in bovine^50^ and rabbit corneal endothelial cells^51^.

Even though TER values using TGF-β1 alone or TGF-β1 plus AG-1478 were generally similar, we observed that ZO-1 and N-cadherin were more localized on the cell-cell junctions in the presence of AG-1478. When TGF-β1 alone was added to the medium, these proteins were distributed at the cell-cell junctions as well as in the cytoplasm. The ZO-1 signal was also weaker in cell junctions in the TGF-β1 alone condition (Fig. 5a and b). Formation of good cell-cell junctions is necessary for the barrier function of the *in vitro* monolayer of HCECs^52^, but the differences observed using protein immunostaining did not have a significant impact on their functionality.

Culture of HCECs using two different media is a new cell culture approach that has been shown to promote the maintenance of their endothelial phenotype for several passages^28,29^. Using a dual media approach, researchers have reported a decrease in the transcription of genes related to proliferation, wound healing and regeneration as well as an upregulation of genes related to the functionality of HCECs^28,29^. Growing cells in a proliferation medium containing growth factors and then stabilizing them for 7 days before passaging in a maintenance medium allows cells to retain their endothelial morphology more consistently than using only a proliferating medium^28,29^. Even if the media formulations were not the same as the ones used in the present study, we reproduced similar results when adding TGF-β1 and AG-1478 to maturation medium. These additives will help obtain cells that show a morphology and function more similar to the native corneal endothelium. However, further studies are required to better understand the effect of TGF-β1 on the phenotype of proliferating and confluent cells as well as its interactions with the growth factors added to the proliferation medium.

Finally, we separated cell populations from Figs 1 to 4 into two categories as follows: low basal expression and high basal expression of α-SMA and type-I collagen. We wanted to see if cells that already showed more signs of EndMT (expression of α-SMA and type-I collagen) would react differently to TGF-β1, a known inducer of EndMT^20^, than cells that expressed fewer signs of EndMT. Many individual factors could explain the differences in α-SMA and type I collagen expression observed between the populations in the present study, e.g., donor age, percentage of viable cells obtained after initial extraction and freezing/thawing before use in experiments. However, cells responded the same to the addition of TGF-β1 during maturation by forming mature cell junctions (Fig. 4b-c) and showing an improved morphology when compared to control cells matured without TGF-β1. The main differences remained in the amplitude of the increases in α-SMA and type I collagen expression when cells were exposed to TGF-β1. HCECs are heterogeneous when cultured, and the rate of success of the cultures is not always high^11,53^, which may partially explain why researchers struggle to establish defined culture conditions that are reproducible from one experiment to another^5,10^.

It is not completely understood how TGF-β1 can promote the cell barrier function of HCECs. *In vivo*, TGF-β2 present in the aqueous humour participates in the maintenance of the non-replicative status of contact-inhibited HCECs^32^. Perhaps TGF-β1 generates a similar effect on HCECs during maturation phase. It would make sense that cells first need to stop cycling before developing mature cell junctions. Further studies could help understand this phenomenon.

In conclusion, this study showed that adding TGF-β1 to the culture media of confluent HCECs was an efficient way to enhance endothelial morphology and obtain functional cell monolayers. This study also elucidated the different roles of TGF-β1 on HCECs and EndMT, representing forward progress in establishing ideal HCECs culture conditions to reconstruct a corneal endothelium that could be used as an alternative treatment for patients affected by endotheliopathies.

## Methods

All experiments were conducted in accordance with the Declaration of Helsinki. The research protocol was approved by the *“Bureau de l’éthique de la recherche du CHU de Québec – Université Laval” ethics committee* (DR-002-1382).

### Cell isolation and culture

Ten cadaveric corneas, non-suitable for human transplantation, were obtained from our local eye bank (Centre Universitaire d’Ophtalmologie (CUO) Eye bank, Québec, Qc, Canada). Donor age ranged from 20 to 71 (49.2 ± 19.1 years old). Corneal endothelial cells were isolated and cultured as previously described^54^. Briefly, CECs and Descemet membrane were peeled from the whole cornea. The strips were incubated in 0.02% EDTA solution at 37 °C for 1 h to loosen cell-cell junctions. Finally, CECs were detached from Descemet membrane by gentle “up-and-down” in a glass pipette and then CECs were seeded on plastic dishes coated with FNC coating mix (AthenaES, Baltimore, MD). All cell populations were used at passage 2 or 3 and plated at 20 000 cells/cm², unless otherwise noted.

Cells were analysed following two culture phases. For the proliferation phase, cells were grown until they reached confluency. The basal proliferation medium (P-medium) consisted of Opti-MEM-I medium (Life Technologies, Burlington, Ontario, Canada) supplemented with 8% fetal bovine serum (FBS; Life Technologies), 5 ng/ml epidermal growth factor (EGF; Austral Biologicals, San Ramon, CA), 1,6% chondroitin sulfate (Sigma-Aldrich, Oakville, Ontario, Canada), 20 µg/ml ascorbic acid (Sigma-Aldrich) and penicillin/streptomycin (VWR, Radnor, PA). For the maturation phase, cells were kept in culture for 7 or 14 days post-confluency. The basal maturation medium (M-medium) consisted of Opti-MEM-I (Life Technologies), 8% FBS (Life Technologies) and antibiotics as described previously. Medium was changed every 2 to 3 days.

### Effect of TGF-β1 on proliferating and mature HCEC

Experiments were performed using four different cell populations. Proliferation phase: HCECs were cultured in the P-medium alone (control) or supplemented with 2ng/ml TGF-β1 (CHO-cell derived, R&D Systems, Minneapolis, MN), a combination of TGF-β1 (2ng/ml) and SB431542 (10 μM)(SB 431542 hydrate, Sigma-Aldrich), a specific type I TGF-β receptor (ALK5) kinase activity inhibitor, or a combination of TGF-β1 (2 ng/ml) and AG-1478 (1 μM)(AG-1478 hydrochloride, Tocris Bioscience, Bristol, UK), a competitive inhibitor of epithelial growth factor receptor. Maturation phase: HCECs were cultured in the P-medium until confluency, then switched to the M-medium alone (control) or supplemented with TGF-β1 (2 ng/ml), a combination of TGF-β1 (2 ng/ml) and SB431542 (10 μM), a combination of TGF-β1 (2 ng/ml) and EGF (5 ng/ml), or a combination of TGF-β1 (2ng/ml), EGF (5ng/ml) and AG-1478 (1 μM), for 7 days. Some HCECs were also further cultured for another seven days in the basal maturation medium, without TGF-β1. Culture conditions are summarized in Table 1. Dose-response assessments for TGF-β1, SB431542 and AG-1478 are detailed in the supplementary information file.

### Trans-endothelial resistance and permeability to Dextran-FITC

HCEC (n = 3 populations) were grown on 60 mm² standing culture inserts (EMD-Millipore, Etobicoke, Ontario, Canada) coated with FNC-coating Mix (Athenaes, MJS Biolynx, Inc, Brockville, Ontario, Canada). They were grown until confluency in the P-medium or in the P-medium containing SB431542 (10 μM) and then switched to the M-medium for 7 days post-confluency (Table 2). Measurements of TER were made at confluency and at 1, 3, 5 and 7 days post-confluency. Medium was first changed and TER was measured after letting the fresh medium equilibrate for 30 minutes. A pair of electrodes and a Millicell ERS-2 voltohmeter (EMD Millipore) was used to measure TER. The small electrode was inserted into the culture insert and the long electrode was inserted into the well outside the insert. Five measurements by inserts were made and 3 inserts were used by condition. Inserts without cells containing only the medium were used as blanks to normalize the measurements. The mean and standard deviation of the 15 measurements by condition were calculated.

**Table 2:** Culture conditions of HCECs on standing culture inserts.

Proliferation medium (P)	Maturation medium (M)
P	M
P	M + TGF-*β* 2 ng/ml
P	M + TGF-*β* 2 ng/mlM + AG1478 1 µM
P + SB431542	M
P + SB431542	M + TGF-*β* 2 ng/ml
P + SB431542	M + TGF-*β* 2 ng/mlM + AG1478 1 µM

HCEC: human corneal endothelial cells; TGF-β1: transforming growth factor-1.

At day 7 post-confluency, after measurement of TER, two of the membranes from each condition were fixed in paraformaldehyde 4% (Electron Microscopy Sciences, Hatfield, PA) and used for indirect immunofluorescences against ZO-1 and n-cadherin. The remaining inserts were used for measurements of the cell permeability to dextran-FITC. Briefly, 40 μg/ml Dextran-FITC 4 kDA (Sigma-Aldrich) was added to the medium inside the standing insert. After 2,5 hours of incubation at 37 °C in the dark, the medium under the standing insert was collected. Fluorescence at 530 nm was analyzed with a plate reader (CytoFluor 4000, Life Technologies)(5 readings). Fluorescence from the samples was normalized to the fluorescence from the blank insert.

### Indirect Immunofluorescence

Immunofluorescence analysis was conducted on cells cultured on glass coverslips and on insert-membranes. Briefly, cells were rinsed with phosphate-buffered saline (PBS), then fixed with 4% paraformaldehyde (Electron Microscopy Sciences) for 10 minutes at room temperature and rinsed 3 times with PBS. Cells were permeabilized with 0,2% Triton X-100 (Fisher Scientific, Ottawa, Ontario, Canada) for 10 minutes before blocking with 10% FBS diluted in PBS for 30 minutes. Cells were incubated with the primary antibodies: a mouse monoclonal anti n-cadherin (BD Biosciences, Mississauga, Ontario, Canada, 1/300), a mouse monoclonal anti type I collagen (Sigma-Aldrich, 1/1000), a mouse monoclonal anti smooth muscle actin (Dako, 1/400), a mouse monoclonal anti ZO-1, (Life Technologies, 1/100), or Alexa fluor 488 conjugated Phalloidin (Life Technologies, 1/200). Primary antibodies were diluted in PBS-FBS 2% and incubated for 1 hour in the dark. Cells were rinsed with PBS, PBS-FBS 2% and PBS, then incubated with the secondary antibody (anti-mouse IgG Alexa Fluor 594, 1/400) and Hoechst 33258 (1/100) diluted in PBS-FBS 2% for 1 hour in the dark. Coverslips were mounted on glass slides with mounting medium (glycerol, PBS, water, gelatin and sodium azid) and kept at 4 °C until observation. Micrographs were acquired using a fluorescence microscope (Zeiss Axio Imager.Z2) and AxioVision 4.8.2 software. Determination of the ratio of the area covered by α-SMA and total actin was made using Image J software. A threshold value was determined for each of the markers using Otsu method and applied to the micrographs. The area covered by the markers was then obtained by the “Analyse particles” function. The number of cells expressing type I collagen was calculated using Image J and the number of total nuclei was obtained using Cell Profiler software. A threshold value for the nuclei was determined with the Otsu method, with a correction factor of 1.1 and a diameter to detect between 10 and 40 pixels. Three micrographs for each of the two or three coverslips per culture conditions were analysed.

### Immunoblotting

Total proteins were extracted with RIPA 1X buffer containing cocktails of phosphatase inhibitors (Halt phosphatase inhibitor, Life Technologies) and protease inhibitors (Complete protease inhibitor, EDTA-free, Life Technologies), as well as phenylmethanesulfonyl fluoride (PMSF) (Cell signalling,Technology, Danvers, MA). Extracts were kept on ice for 30 minutes, then centrifuged at 14 000 RPM at 4 °C for 30 minutes. The supernatants were then collected and kept at −80 °C. Protein concentration was determined using a micro-BCA protein assay kit (Pierce BCA protein assay, Life Technologies) according to the manufacturer’s instructions. Proteins were diluted at 0,5 µg/ml using 4 × Laemmli buffer (Bio-Rad) and denatured at 95 °C for 5 minutes. Samples were run on 7,5 or 10% acrylamide gels (19:1 30% acrylamide/bis-acrylamide solution, Bio-Rad) at 120 V and transferred overnight on 0,45 μM nitrocellulose membranes (Bio-Rad) at 4 °C and 30 V. Membranes were blocked in 5% non-fat powdered milk diluted in tris-buffered saline (TBS) containing 0,5% tween-20 (Promega, Madison, WI) for one hour. Primary antibodies (mouse monoclonal anti-smooth muscle actin (1/2000) (Dako); mouse monoclonal anti-n-cadherin (1/2000) (BD Biosciences)) were diluted in 5% non-fat powdered milk in TBS-tween and were added to the membranes for 1 h on a shaking platform. After rinsing with TBS-tween for 30 minutes, membranes were incubated with the secondary antibody (1/2000) (peroxydase-conjugated Affini-Pure goat anti-mouse antibody, Jackson Immunoresearch) for 1 h on a shaking platform. Membranes were revealed using enhanced chemiluminescent substrate (Western Sure Premium Chemiluminescent Substrate, Li-COR, Lincoln, NE) and a blot scanner (C-Digit Chemiluminescence Western Blot Scanner, Li-COR). Tubulin served as loading control (1/5000)(mouse anti α-tubulin antibody; Abcam, Toronto, Ontario, Canada). Relative quantification of the western blots was made using Image Studio Digits software (Li-COR). Mean of the measurements for each sample and standard deviation were calculated using Microsoft Excel software. Results are presented as % of the protein relative to the loading control for 4 populations, with the exception of the results in Fig. 2e (2 populations analysed).

### Statistical analysis

Results are presented as mean of all measurements and standard deviation (SD). Statistical significance was calculated with one or two-way ANOVAs, followed by Tukey’s multiple comparisons test using GraphPadPrism©. A p < 0,05 was considered significant.

## Electronic supplementary material

Supplementary data
