# Supplementary material for: TGF-β1 promotes cell barrier function upon maturation of corneal endothelial cells
Source: Sci Rep. 2018 Mar 13;8:4438. doi: 10.1038/s41598-018-22821-9 (PMC5849742; doi:10.1038/s41598-018-22821-9)
Supplement: Supplementary file 1 — Supplementary data [file 41598_2018_22821_MOESM1_ESM.pdf]

TGF-β1 promotes cell barrier function upon maturation of corneal endothelial cells.

AUTHORS: Véronique Beaulieu Leclerc[†, 1-2], Olivier Roy[†, 1-2], Kim Santerre[1-2] and Stéphanie Proulx*, 1-2

[†]equal contributors

AFFILIATIONS:

1. Centre de recherche du Centre hospitalier universitaire (CHU) de Québec – Université Laval, axe médecine régénératrice, Hôpital du Saint-Sacrement, Québec, QC, Canada;
2. Centre d'organogénèse expérimentale de l'Université Laval/LOEX, Québec, QC, Canada and Département d'Ophtalmologie, Faculté de médecine, Université Laval, Québec, QC, Canada.

*Correspondence and requests for materials should be addressed to S.P.
stephanie.proulx@fmed.ulaval.ca

Supplementary data S1

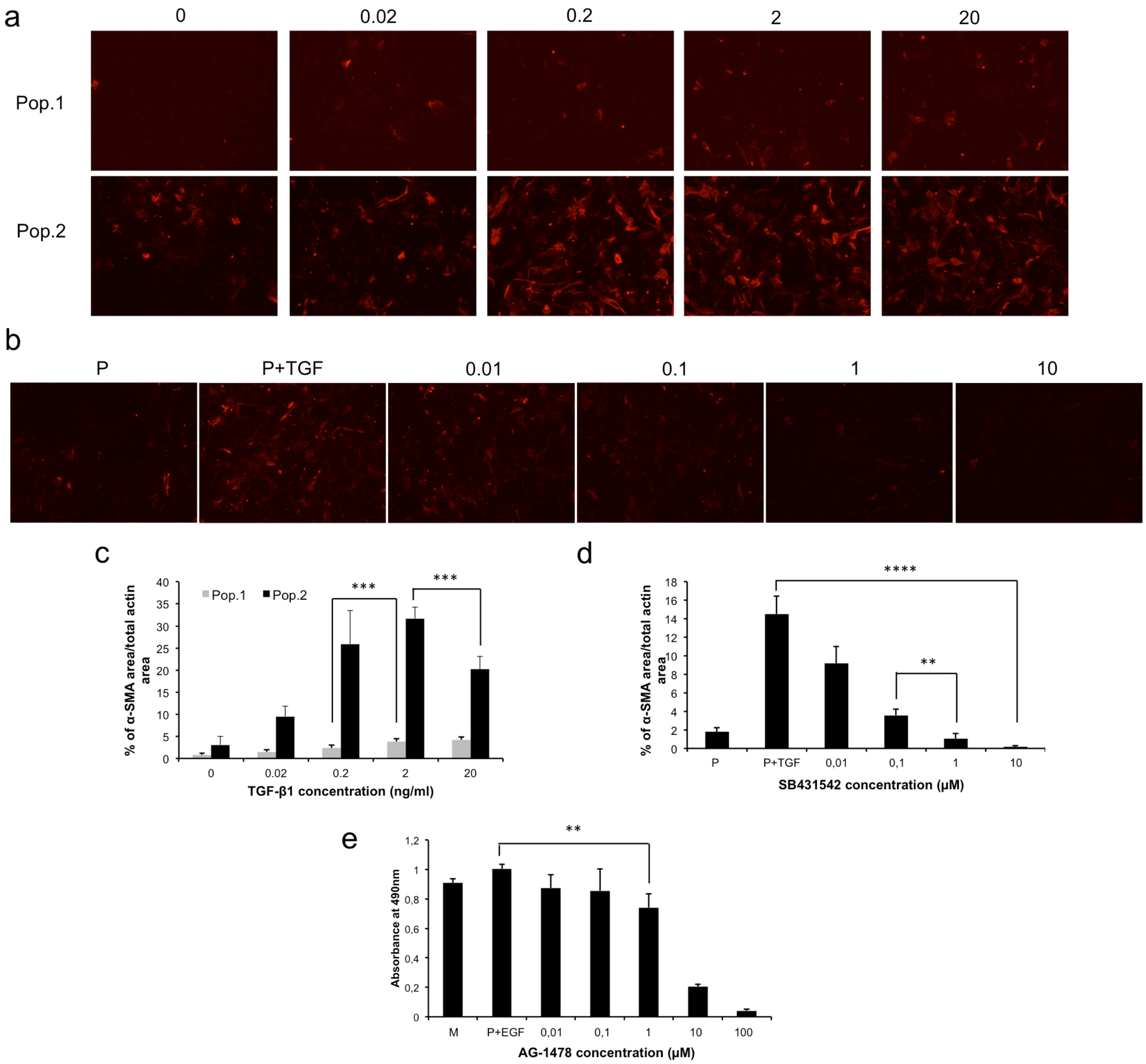

Supplementary figure S1: Dose-response assessments for TGF-β1, SB431542 and AG-1478

a) Dose-response assessment for TGF-β1. Immunofluorescence detection of α-SMA in CECs cultured in the basal proliferating medium (P-medium) and with increasing TGF-β1 concentrations until confluency, Immunofluorescence was performed at confluency.

b) Dose-response assessment for SB431542. Immunofluorescence detection of α-SMA in CECs cultured in the basal proliferating medium (P-medium), with P-medium containing 2 ng/ml TGF-β1 and with increasing SB43152 concentrations, an inhibitor of type I transforming growth

factor receptor. Cells were cultured until confluency and immunofluorescence was then performed.

c) Dose-response assessment for TGF-β1. Analysis of the ratio of the area covered by α-SMA and total actin in two different populations of HCECs (grey bars: Population 1; black bars: population 2) depending of the TGF-β1 concentration in the culture medium. One-way ANOVA followed by Tukey's multiple comparison tests were performed. Results are presented as mean ± standard deviation for each condition and population.

d) Dose-response assessment for SB431542. Analysis of the ration of the area covered by α-SMA and total actin in one population of HCECs depending of the SB431542 concentration in a culture medium containing 2ng/ml TGF-β1 (P+TGF). One-way ANOVA followed by Tukey's multiple comparison tests were performed. Results are presented as mean ± standard deviation for each condition.

e) Dose-response assessment for AG-1478. Analysis of absorbance following a MTS proliferation assay in cells cultured in a basal proliferation medium (P+EGF) and exposed to growing AG-1478 concentrations for 72h. One-way ANOVA followed by Tukey's multiple comparison tests were performed. Results are presented as mean ± standard deviation for each condition.

**p<0.005; ***p<0.0005; ****p<0.0001

α-SMA: α-smooth muscle actin; TGF-β1: transforming growth factor β-1; EGF: epidermal growth factor.

Supplementary methods S1

Dose-response assessments for TGF-β1, SB431542 and AG-1478. HCECs (n=2 populations, 2 coverslips/condition) were seeded at 20 000 cells/ cm² and cultured in the basal proliferation medium (P)(see main text, methods section) supplemented with increasing concentrations of TGF-β1 (0, 0.2, 2, 20 ng/ml) (recombinant human transforming growth factor beta 1, R&D systems, Minneapolis, MN) until confluency (4-5 days). Double immunofluorescence for actin (Life Technologies) and α-smooth muscle actin (α-SMA; Dako, Burlingtion, Ontario, Canada) was performed as described in the indirect immunofluorescence paragraph. Micrographs (3/coverslip, 2 coverslips/TGF-β concentration) were acquired using a fluorescence microscope (Zeiss Axio Imager.Z2, Carl Zeiss, Toronto, Ontario, Canada) and AxioVision 4.8.2 software. Determination of the ratio of the area covered by α-SMA and total actin was made using Image J

software. Mean ratio and standard deviation were calculated for each concentration. The ideal dose was determined by the concentration that induced higher area of α-SMA without inducing cell mortality.
A similar protocol was used to determine the ideal concentration of SB431542, an inhibitor of type I transforming growth factor receptor (TGF-beta RI Kinase Inhibitor, SB431542 hydrate, Sigma-Aldrich). After determining the ideal concentration of TGF-β1, HCECs (n=1 population, 2 coverslips/condition, 3 micrographs/coverslip) were cultured in basal proliferation medium (P) containing 2 ng/ml of TGF-β1 and supplemented with SB421542 (0, 1, 10 and 100 μM) until confluency. Immunofluorescence against α-SMA and total actin was analyzed using image J software as described in the preceding paragraph. The ideal dose was determined by the concentration that blocked the most the expression α-SMA induced by TGF-β1 without inducing cell mortality.

Dose-response to AG-1478 hydrochlodride (Tocris Bioscience, Minneapolis, MN), an inhibitor of EGF receptor, was made using a MTS proliferation assay (CellTiter 96® Non-radioactive Cell Proliferation Assay, Promega, Madison, WI). Briefly, one primary population of HCECs (4 wells per condition) was plated onto 96 wells-plates at a seeding density of 15 000 cells/cm^2 in the basal maturation medium (M). After 24h of adhesion, medium was changed to a basal proliferation medium (P) supplemented with EGF 5 ng/ml and increasing concentrations of AG-1478 (0.01, 0.1, 1, 10 and 100 μM). After 72h of culture, 20 μl of MTS reagent was added to the medium. After 2h of incubation in the dark, absorbance at 490 nm was acquired using a microplate reader (Bio-Rad Model 550 microplate reader, Mississauga, Ontario, Canada) and Microplate Manager 5.0 software. Mean absorbance and standard deviation were calculated for each concentration of AG-1478 (4 wells per condition). The ideal concentration was determined by the concentration that blocked the most proliferation induced by EGF without inducing cell mortality.

Statistical analysis: Results are presented as mean of all measurements and standard deviation (SD). Statistical significance was calculated with one-way ANOVAs, followed by Tukey's multiple comparisons test using GraphPadPrism©. A $p<0,05$ was considered significant.

Supplementary results S1

Dose-response assessments to TGF-β, SB431542 and AG-1478. Ideal doses of TGF-β1, SB431542 and AG-1478, 3 agents added to the medium of our different experiments, were first

assessed with dose-response assays. Response to various concentrations of TGF-β1 and SB431542 was evaluated by the ratio of the area covered by α-SMA on immunofluorescence micrographs to the area covered by total actin.

The TGF-β1 ideal concentration was determined to be 2 ng/ml (fig.S1a and c) because it induced the most important expression of α-SMA in both cell population that were tested. It was higher than with 0.2 ng/ml, although not statistically different for population 2. Cells proliferated less in 20 ng/ml of TGF-β1 (experimenter's observation). 10 µM of SB431542 was sufficient to totally block expression of α-SMA induced by 2 ng/ml TGF-β1 (fig.S1b and d). 100 µM of SB431542 was also tested, but cells poorly proliferated and could not reach confluency in the same time as with the other concentrations. Response to AG-1478 was evaluated by a cell proliferation assay (MTS assay)(fig.S1e). The absorbance at 490 nm correlates with the number of living cells at 72h of culture with AG-1478. Cells had a high mortality in 10 and 100µM of AG-1478 (experimenter's observations). 1 µM was then determined as the concentration of AG-1478 that reduced the most proliferation induced by 5 ng/ml of EGF, without inducing cell mortality.

Supplementary data S2

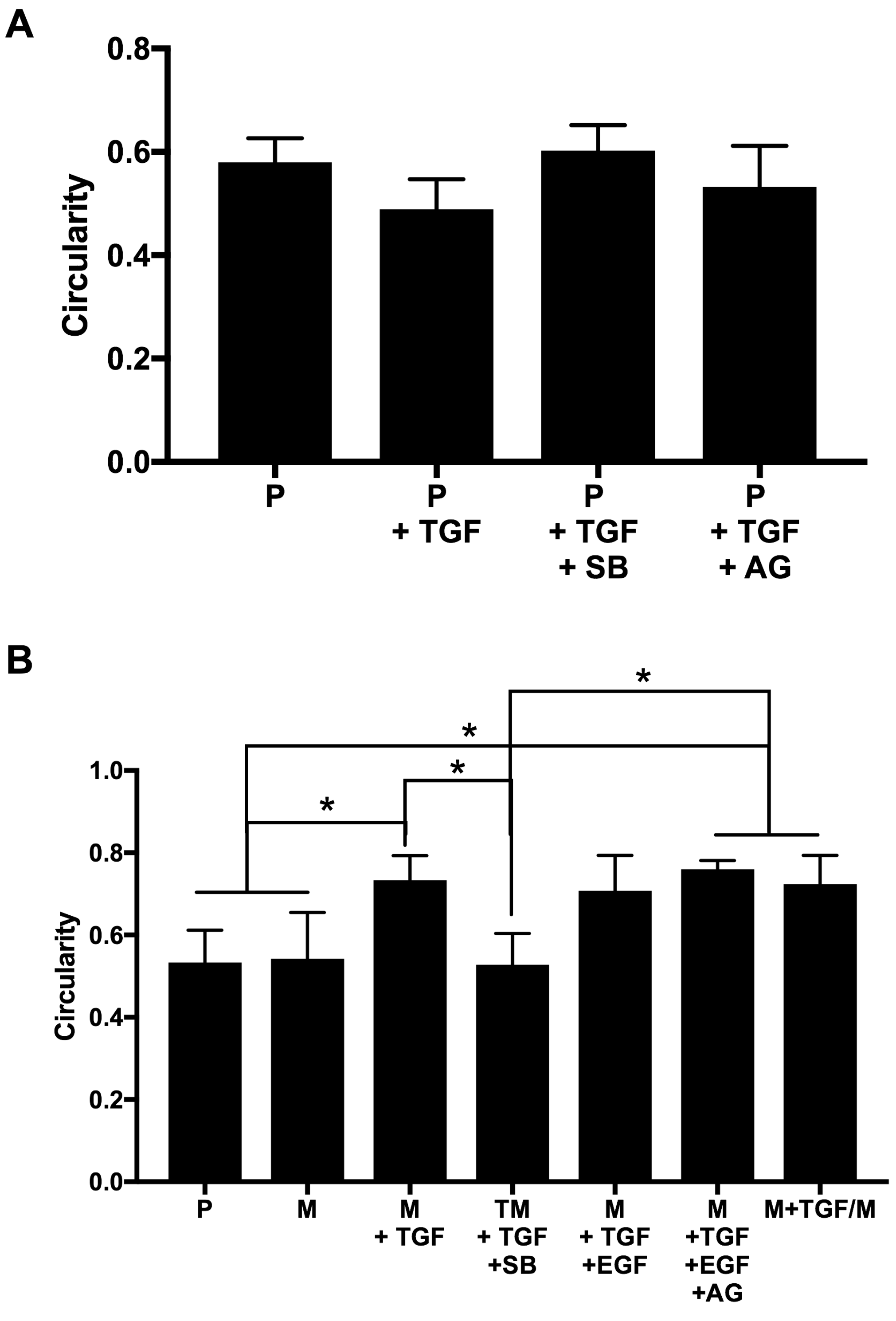

Supplementary figure S2: Measurement of cell circularity.

a) Circularity of HCECS after the proliferation phase. Cells were grown until confluency in a proliferation medium containing 5 ng/ml of EGF plus 2 ng/ml TGF-β1, 10 µM SB431542 or 1 µM Ag-1478**.** One-way ANOVA followed by Tukey's multiple comparison tests were performed. Results are presented as mean ± standard deviation for each condition.
b) Circularity of HCECS after the maturation phase. Cells were grown until confluency in a proliferation medium containing 5 ng/ml of EGF (P). They were then matured for 7 days in a maturation medium (M) and various additives. Cells in the M+TGF/M condition were matured for 7 days in M+TGF-β1 then for another 7 days in the M medium only (a total of 14 days of maturation). One-way ANOVA followed by Tukey's multiple comparison tests were performed. Results are presented as mean ± standard deviation for each condition.

* $p<0.05$

Supplementary methods S2

HCECs (n=4 populations, 2 or 3 coverslips per condition, 3 micrographs per coverslip) were cultured as described before in different proliferation and maturation media (see table 1 in the main article). Phase contrast images (0 and 7 or 14 days post-confluency) were used to define cell circularity. At least 100 randomly selected cells per condition were measured. Their area and perimeter were acquired with ImageJ software and then circularity was determined by the formula:

$$Circularity = 4\pi\left(\frac{Area}{perimeter^2}\right)$$

Hexagonal cells, an hallmark of endothelial morphology, have a circularity close to 1.0 and cells with fibroblastic morphology get a score closer to zero. Statistical difference between the results was calculated with Tukey's multiple comparison test, where $p<0.05$ was considered statistically significant.

Supplementary results S2

Measurement of cell circularity after the proliferation (fig. S2a) (0 days post-confluency) show that addition of TGF-β1 to the medium tended to lower the circularity, compared to the control P medium. The negative effect of TGF-β1 was also neutralised by the addition of SB431542, were the circularity is similar to the P control. However, these results are not statistically significant.

After 7 days of maturation in different additives, there were statistically significant differences in the cell circularity. First, addition of TGF-β1 to the maturation medium improved circularity when

compared to the maturation control (M) (0.7338 vs 0.5425, p=0.02). Circularity was also higher than maturation control in TGF-β1 + EGF + AG-1478 and the 14 days post-confluency conditions. Finally, addition of SB431542 to the medium counteracted the beneficial effect of TGF-β1 to the circularity. Circularity was similar to the control maturation condition and lower than all of the TGF-β1 containing conditions. This confirms the positive effect of TGF-β1 on cell circularity of maturing HCECs and shows that it can improve endothelial morphology.

Supplementary data S3

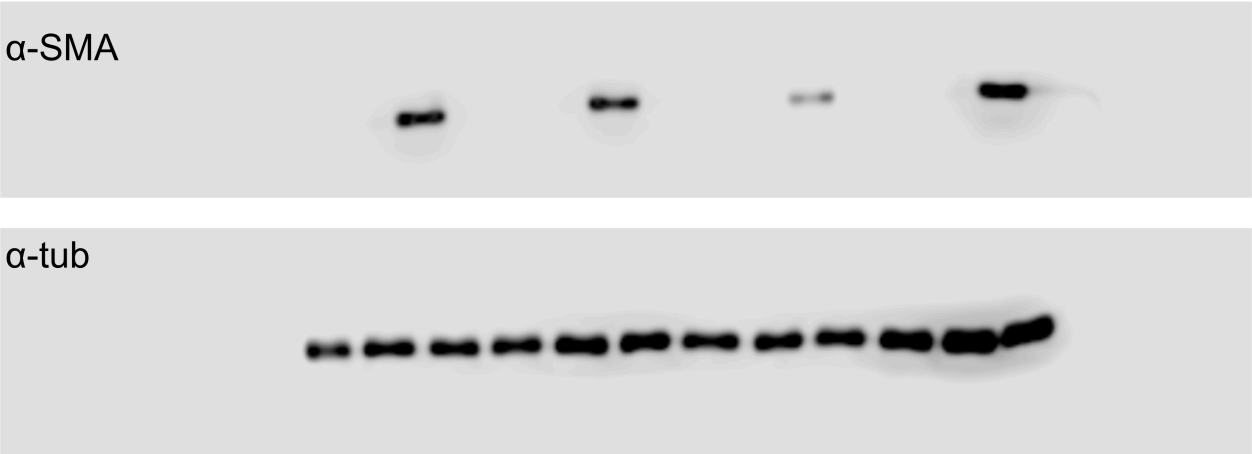

Supplementary figure S3: Full-length blot of Fig. 1f

Supplementary data S4

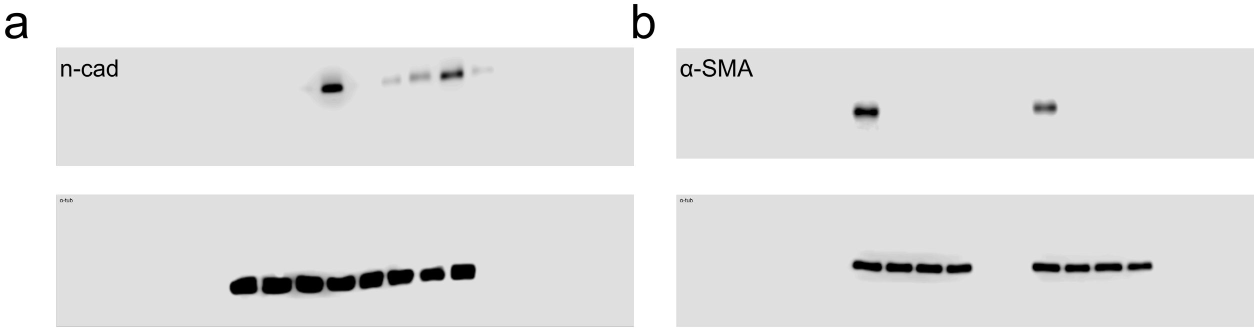

Supplementary figure S4: Full-length blot of Fig. 2b (a) and Fig. 2e (b)

Supplementary data S5

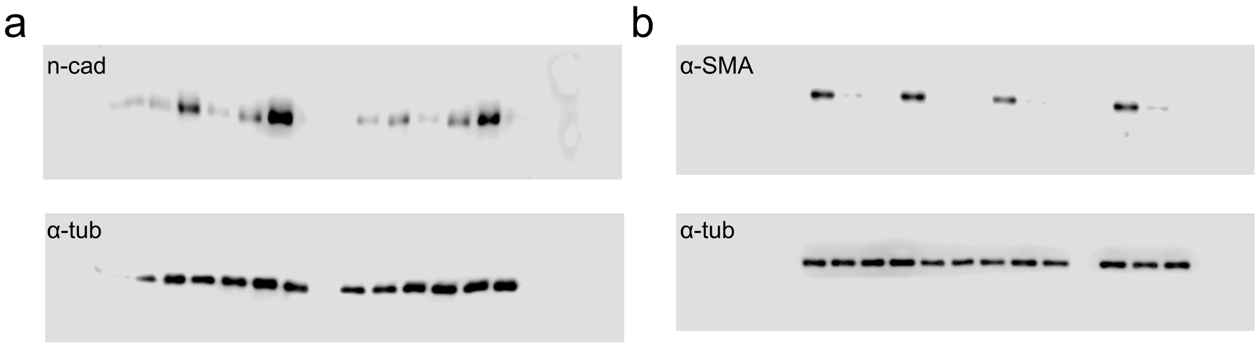

Supplementary figure S5: Full-length blot of Fig. 3b (a) and Fig. 3e (b)

Supplementary data S6

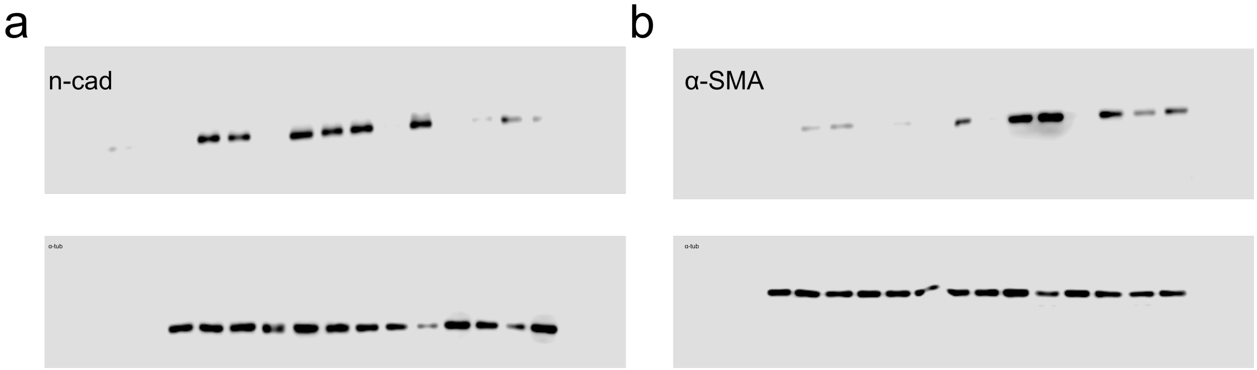

Supplementary Figure S6: Full-length blot of Fig. 4c (a) and Fig. 4f (b)
